# SURVEY STUDY: SPORTS INJURIES IMPACT ON MENTAL HEALTH, ACADEMICS, AND INTERPERSONAL RELATIONSHIPS IN COLLEGIATE ATHLETES

**DOI:** 10.51894/001c.123029

**Published:** 2024-08-30

**Authors:** Landon Barlow

**Affiliations:** 1 College of Osteopathic Medicine Michigan State University

## 58

### INTRODUCTION/BACKGROUND

Being an athlete is associated with the inherent risk of sport-related injuries. Each year thousands of athletes in the United States will sustain an injury while competing in their respective sport(s). This can have a profound impact on their physical and mental well-being. This study seeks to explore and quantify the repercussions of these injuries on collegiate athletes’ mental health, academics, and interpersonal relationships.

### OBJECTIVES/HYPOTHESIS

Current Literature underscores the importance of effective treatment for athletes’ musculoskeletal health and overall well-being. The researchers in this study hypothesize that the severity and duration of sports injuries in collegiate athletes will directly correlate with negative consequences in the domains of mental health and social well-being.

### METHODS

This cross-sectional study utilizes an IRB-approved survey consisting of thirty-one questions made up of multiple choice, text entry, sliding scales, and Likert scales, to evaluate the parameters and set out in the study. Data collection is being gathered through an online survey platform. The survey covers a range of topics, including demographic information, injury history, mental health indicators, academic performance, and details about interpersonal relationships. Descriptive statistics, inferential statistics, multivariate analysis, regression analysis, chi-square, and, finally, logistic regression, will be employed to evaluate the dependent variables in the study.

### RESULTS

This study is currently in data collection with final analysis in the coming weeks. Initial review from respondents has been quite remarkable. We anticipate no issues having meaningful results.

### DISCUSSION/CONCLUSION

This research seeks to explore the social and mental well-being of collegiate athletes who have sustained a sports-related injury during their collegiate career. It aims to expand existing research in this domain with an emphasis on collegiate athletes to educate athletes and their stakeholders on the potential effects injuries have on the social and mental well-being of this population.

